# Escape from self: Stress increase consumers' preference for experiences over material possessions

**DOI:** 10.3389/fpubh.2022.1070947

**Published:** 2023-01-05

**Authors:** Yurou Zhao, Xiaotong Jin, Taiyang Zhao, Jianan Li

**Affiliations:** ^1^School of Business and Management, Jilin University, Changchun, China; ^2^School of Philosophy and Sociology, Jilin University, Changchun, China

**Keywords:** stress, self-escape, experiential consumption, material consumption, cognitive resources

## Abstract

**Introduction:**

Considering the theory of self-escape, this research systematically investigates the effect of stress on consumers' preference for experiences vs. material possessions.

**Methods:**

We conducted one survey and two experiments to demonstrate the effect of stress on individuals' relative preference for experiential vs. material consumption and its psychological mechanism.

**Results:**

The findings of the three studies revealed that stress increases consumers' preference for experiences over material possessions. Additionally, self-escape motivation plays a mediating role between stress and preference for experiential consumption (vs. material consumption). Stress as a self-threat triggers individuals' motivation to escape from negative self-concept, and experiences can help individuals temporarily escape from negative self-recognition and provide more leisure value than material possessions. Therefore, individuals increase their consumption preference for experiences. Furthermore, we observed that the type of experiences plays a moderating role between stress and preference for experiential consumption (vs. material consumption). Specifically, compared with low cognitive resource demanding experiences, the effect of stress on experiential consumption disappears when experiences have a high demand for cognitive resources.

**Discussion:**

These findings extend the research on stress, experiential consumption and material consumption and provide significant advice for public mental health.

## 1. Introduction

Stress has become a common psychological state of modern individuals. According to data from Gallup's sentiment survey in 2021, the global stress level reached an all-time high–40%. Stress is a negative psychology that propels individuals to cope in various ways. One common way to deal with stress is through consumer behavior. Exiting research has explored the impact of stress on material products, such as luxury goods (1–3) and unhealthy foods (4), few studies have focused on the impact of stress on experiential products. A survey of office workers from China revealed that ~70% would prefer to travel as their main choice when experiencing stress. In addition, the survey report highlighted that 12% would prefer to watch a concert, and 10% would prefer to watch a movie, reflecting that stressed individuals desire experiential consumption, which provides a new idea for relevant research. However, the relationship between stress and consumption preference for experiences remains unclear. Considering that experiential consumption and material consumption are relative concepts in the field of consumption (5), we raise the following questions based on the above phenomenon and research gaps: Does stress affect an individual's consumption preference for experiences and material possessions? What is the psychological mechanism between them? Does the effect always exist?

Our study introduces the theory of self-escape to explore these issues. Previous studies have found that stress immerses an individual's mind in stressful situations, thereby enhancing the individual's persistent belief that they are unable to cope with environmental demands and reinforces the individual's negative self-perception. In this case, individuals will have a predisposition tendency to avoid self-perception, i.e., self-escape motivation, as a result of their self-defense instinct. Compared with material consumption, experiential consumption can help individuals divert their attention, and temporarily separate from stressful situations, thereby reducing negative self-perception. Consequently, individuals will increase their consumption preference for experiences. By examining the above-mentioned aspects, our study clarifies the relationship between stress and consumption preference for experiences and material possessions, and further reveals that self-escape motivation acts as a significant factor influencing the consumption of experiences. Finally, the paper concludes with the identification of suggestions for public mental health and puts forward prospects for future research in related fields.

## 2. Theoretical framework

### 2.1. Stress

Stress is an emotional state frequently experienced by individuals; it generally refers to a state of psychological tension that individuals experience in work, life, interpersonal relationships, and personal responsibilities. It can occur when individuals perceive their environmental demands to be taxing or beyond their available coping resources, thereby endangering their overall wellbeing (6). As a negative psychological state, stress motivates individuals to undertake various coping measures (7, 8). Using consumption to cope with stress has become relatively common. For example, previous studies have deduced that in response to stress, consumers may have a stronger preference for compulsive purchases and impulsive purchases (9) to relieve stress and negative emotions (10), enhance excitement and pleasure (11), and evoke hedonism (12). In addition, research suggests that stress increases individuals' consumption preference for nostalgic products. By recalling people or events in the past through nostalgic consumption, individuals can maintain positive self-evaluation and social support, thereby increasing positive emotions and alleviating current stress (13). Furthermore, previous literature has shown that when consumers feel stressed, their consumption behaviors are aimed at reducing the negative emotional distress and improving their emotions through consumption. Therefore, our study proposes that the consumption of experiences may also help consumers achieve the same purpose.

### 2.2. Stress and consumption preference for experiences vs. material possessions

Experiential and material consumption are two relative concepts (5). Experiential consumption refers to the consumption performed with the primary intention of acquiring life experiences, emphasizing “process and experience,” such as traveling and going to theme parks (14). Material consumption refers to the consumption performed with the primary intention of obtaining material possessions, emphasizing “preservation and possession,” such as electronic products. Previous studies have predominantly focused on the distinction between the outcome variables of experiential and material consumption. For example, compared with material consumption, experiential consumption is perceived to be more unique (15), more able to assist individuals develop their ideal-self (16), and offers greater happiness to individuals (1–3). Conversely, material consumption is more likely to be recognized by others than experiential consumption; therefore, it can satisfy the material and symbolic needs of individuals (17). However, limited studies have focused on the factors that affect individuals' relative preferences for experiential and material consumption.

This study explores the effect of stress on individuals' preference for experiential vs. material consumption. In response to stress, individuals typically cope by pursuing positive emotions and avoiding the interference of negative emotions (18). Prior research has demonstrated that the consumption of experiences can generate more sustained happiness than material possessions (19, 20). As a result of this finding, the consumption of experiences (vs. material possessions) has become an effective strategy that helps individuals relieve the negative effects of stress. Therefore, in response to stress, individuals may be more inclined to undertake experiential rather than material consumption to alleviate negative emotions and achieve leisure. Consequently, we hypothesize the following:

Hypothesis 1: Stress will increase individuals' consumption preference for experiences over material possessions.

### 2.3. Mediating role of self-escape motivation

Self-escape theory describes an individual's desire to escape from negative self-perceptions (21). When an individual has a negative self-perception and cannot resolve it, self-escape motivation occurs (22). Stress can increase an individual's self-escape motivation. From a direct perspective, stress immerses an individual's mind in stressful situations (23), thereby enhancing the individual's persistent belief that they are unable to cope with environmental demands. This reinforces the individual's negative self-perception and increases their cognitive load. Individuals will have a predisposition tendency to avoid self-consciousness, i.e., self-escape motivation, as a result of their self-defense instinct (24). From an indirect perspective, previous studies have determined that stress is often regarded as a threat by individuals, thus triggering anxiety (25). Therefore, emotions are an important signal for interpreting individual motivation and behavior. According to the cognitive-motivational-relational theory of emotion, anxiety reflects an individual's motivation to avoid potential threats and the tendency to flee (26). Whether from a direct or indirect perspective, stress can stimulate an individual's self-escape motivation. Therefore, we hypothesize the following:

Hypothesis 2: Stress increases an individual's motivation to escape from the self.

There are several ways to escape from the self. Existing research has indicated that drinking, overeating, and even suicide are all self-escape mechanisms (27, 28). Therefore, an individual escapes from the self mainly by diverting attention and disengaging from stressful situations. However, these methods negatively impact the individual. In this study, we propose that the consumption of experiences can be regarded as a form of happy escape. Experiential consumption is a consumption pattern wherein individuals engage in experiences (5). Therefore, through experiential consumption, individuals can divert their attention, increasingly focus on experiences (29), and temporarily separate from stressful situations (30), thereby reducing negative self-awareness and creating leisure (31). Previous research has also deduced that stress increases individuals' preference for risky experiences. Consequently, individuals can fully devote their attention to the activity and avoid stress (32). Research about tourism has also found that one of the key motives for individuals to travel is to escape from daily life (1–3, 33, 34). In addition, compared with material possessions, participating in experiences can help individuals construct an ideal-self (16), which increases their positive self-evaluation. Therefore, the dual benefits of reducing negative self-perceptions and building positive self-perception make experiential (vs. material) consumption an effective strategy of helping individuals escape. Thus, the self-escape motivation makes individuals more inclined toward consuming experiences rather than material possessions. Therefore, we hypothesize as follows:

Hypothesis 3: Self-escape motivation increases the individual's consumption preference for experiences (vs. material possessions).

Stress can trigger individuals' motivation to escape from negative self-perception, and experiences can facilitate the temporary escape from negative self-perception and provide more happiness and leisure value than material possessions. Consequently, individuals increase their consumption preference for experiences (vs. material possessions) to achieve temporary leisure and relief. Therefore, we hypothesize the following:

Hypothesis 4: Self-escape motivation plays a mediating role between stress and individuals' consumption preference for experiences (vs. material possessions).

### 2.4. Boundary condition

It is difficult to reduce individuals' negative self-awareness, as self-escape implies both diverting attention and narrowing it to the current environmental stimuli. Moreover, the concept of self-escape further implies avoiding meaningful thinking (35), i.e., a low construal level of attention demand. While most experiences are more relaxing and leisurely than material consumption, there are also unusual cases where the process of experiences is more complicated and consumes more psychological resources (e.g., intellectual games). In this case, experiences that consume significant psychological cognitive resources may aggravate individuals' cognitive burden, strengthen their negative self-perceptions, and deepen the negative impact of stress. Based on these inferences, we propose the boundary condition for the effect of stress on consumption preference for experiences (vs. material possessions)—the type of experiences. Specifically, individuals should only prefer low cognitive resource–demanding experiences that can facilitate the removal of psychological burdens and increase leisure. When experiences have a high demand for cognitive resources, the effect of stress on experiences disappears. Therefore, we hypothesize the following:

Hypothesis 5: The type of experiences plays a moderating role between stress and consumption preference for experiences (vs. material possessions). Compared with experiences with low cognitive resource demands, the effect of stress on consumption preference for experiences disappears when such experiences have high demands for cognitive resources.

### 2.5. Overview of studies

**Figure 1:**
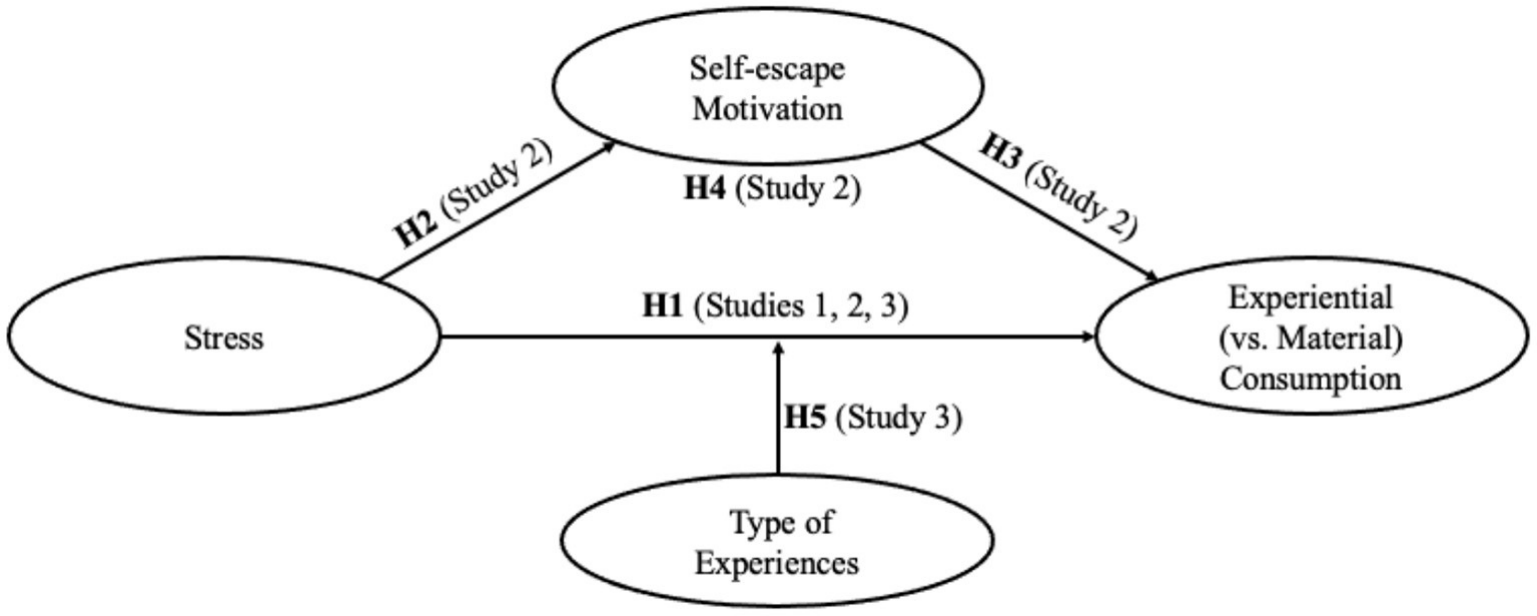
Conceptual framework and outline of the studies and hypotheses.

We conducted three studies to assess our conceptualization of how stress influences individuals' consumption preference for experiences vs. material possessions as well as the boundary conditions discussed above. We measured (Study 1) and manipulated (Studies 2–3) feelings of stress. In Study 1, we measured participants' perceived stress and their consumption preference for experiences vs. material possessions. The study confirms that individuals with high stress levels have a high consumption preference for experiences (vs. material possessions), thereby providing initial support for the main effect of stress on experiential (vs. material) consumption. In Study 2, we replicated the effect of stress on experiential (vs. material) consumption under laboratory conditions. In addition, we manipulated stress and then used the binary choices between experiential products and material products to intuitively reflect participants' consumption preference. Furthermore, we also tested the mediation effect of self-escape motivation. In Study 3, we highlighted a boundary condition: if experiences do not provide escape effect, i.e., when experiences consume more psychological cognitive resources, the effect of stress on individuals' consumption preference for experiential options will disappear. All product choices used in the three studies were pretested. Figure 1 conceptually summarizes the hypotheses and empirical plan.

## 3. Study 1

Study 1 aimed to test our basic hypothesis that stress influences individuals' consumption preferences for experiences vs. material possessions. We used online questionnaires to measure individuals' daily stress levels and relative consumption preferences for experiences vs. material possessions. We expected a direct correlation between increase in stress levels and increase in preferences for experiential consumption over material consumption.

### 3.1. Participants

Study 1 collected 253 valid questionnaires (with a recovery rate of 98.1%) from a professional platform named Credamo. The sample included 147 female participants (58.1%) and 106 male participants (41.9%), aged 19–58 (Mean = 28.3). A significant number of participants had a large share in expenditure, between 2,000 and 3,000 RMB per month (33.2%), and 67.2% of the participants had completed a college or university program.

### 3.2. Methods

When establishing the questionnaire, we selected representative scales to measure the stress and experiential vs. material consumption preference, which were widely used in previous studies. The questionnaire has passed the audit of Credamo, which guaranteed that it would not cause negative psychological effects on participants. As for the consent of participation, only those who agreed and volunteered to participate in surveys will access the questionnaire and corresponding remuneration. At the beginning of the questionnaire, we once again emphasized that “the survey results are only used for academic research, and the personal privacy of participants will be protected. If you agree and are participating voluntarily, start answering questions; if you disagree or are unsure, please exit.”

The online questionnaire survey comprised two parts: daily life survey and consumer preference survey. After reading the questionnaire description and providing their consent, participants first completed the daily life survey. This survey measured participants' daily life stress based on the research of Lee et al. (36), which consists of five items (Cronbach's α = 0.74). The content of the five items was as follows: “My life was very stressful,” “Problems experienced by others put an extra burden on me,” “I have to deal with a lot of problems on a daily basis,” “Relatives or co-workers expected a lot from me,” and “I am worried about a lot of things.” Participants were required to answer to what extent these statements aligned with their actual conditions. All items used a seven-point Likert scale (ranging from 1 = very inconsistent to 7 = very consistent).

Subsequently, participants were required to complete the consumer preference survey. In this section, we measured participants' relative preference for experiential vs. material consumption based on the research of Howell et al. (37), which consists of four items (Cronbach's α = 0.70). The content of four items included “In general, when I have extra money I am likely to buy a material item or a life experience,” “When I want to be happy, I am more likely to spend my money on material goods or activities and events,” and so on. Additionally, all items used a seven-point Likert scale. Finally, we collected participants' demographic information. Each participant who completed the questionnaire received a 5 RMB.

### 3.3. Results

We established a stepwise regression to verify the establishment of the main effect between stress and individuals' consumption preferences for experiences vs. material possessions. In the regression model, the participants' gender, age, average monthly expenditure, and education level were placed into Model 1 as control variables, stress was placed into Model 2 as an independent variable, and experiential (vs. material) consumption preference was the dependent variable. The empirical results revealed that the regression coefficient of stress on experiential (vs. material) consumption preference is significant (β = 0.19, *p* = 0.003), and the delta R-squared of Model 2 on the basis of Model 1 is significant (Δ*R*^2^ = 0.03, *p* < 0.001). This finding implies that stress had a significant positive effect on participants' preference for experiential (vs. material) consumption. That is, the greater stress experienced by participants, the more they prefer experiences over material possessions. Thus, H1 was verified. Additionally, we also found a positive correlation between monthly average expenditure and experiential (vs. material) consumption preference (β = 0.17, *p* = 0.010), and a negative correlation between educational level and experiential consumption preference (β = 0.16, *p* = 0.016). Moreover, age and gender had no significant effect on the participant's consumption preference (*p* > 0.1).

### 3.4. Discussion

The results of Study 1 showed that individuals' stress levels are positively correlated with their consumption preference for experiences (vs. material possessions), which provide preliminary empirical support for our hypothesis. However, Study 1 has several limitations. First, the stress measurements in this study are calculated from participants' indirect feedback, rather than participants' direct and subjective stress; thus, there may be content deviations. Second, as this study was conducted in the form of online questionnaires, the results of regression analysis can only prove the correlation between the two variables, making it difficult to determine the causal relationship between stress and individuals' consumption preference for experiences (vs. material possessions). Therefore, in Study 2, we will conduct an experiment to solve these problems.

## 4. Study 2

Study 2 aimed to replicate Study 1 in a controlled laboratory environment to establish a robust causal relationship between stress and consumption preferences for experiences vs. material possessions and test the mediation effect. We manipulated participants' stress, and then measured participants' choices between five pairs of experiential and material products and self-escape motivation to examine the relationship between stress and experiential (vs. material) consumption preferences.

### 4.1. Participants

Participants comprised 102 undergraduate students recruited from a large university in China (56 males, 46 females; M_age_ = 19.77). Each participant received 10 RMB as compensation.

### 4.2. Procedure and stimuli

We conducted the experiment in a lab. Participants were told to complete two separate tasks. The first was a recall task testing their memory, which we in fact used to manipulate stress. The second was a hypothetical shopping task, which we in fact used to measure preference for experiences vs. material goods. The participants were blind to the aims of both tasks.

#### 4.2.1. Stress manipulation

First, the participants completed an event recall task to manipulate their stress levels. All participants were instructed that the study's purpose was to collect information regarding general life events. They were asked to write about one type of event, to which they would then be assigned randomly. In the stress condition, the participants were asked to describe a stressful situation they had experienced, whereas in the control condition, they were instructed to write about a typical school day.

#### 4.2.2. Dependent variable

Following completion of the event recall task, participants' preference for experiences vs. material goods was measured using the method adopted from the study conducted by Tully et al. (38). Participants were instructed to choose between five pairs of products with the same price, each consisting of one “experience” and one “material good.” For each pair, the participants indicated their preferred products using a binary choice. We randomized both the order of the five pairs and the position of the “material good” and “experience” within each pair. The same randomization procedure was followed in subsequent studies. Additionally, we conducted a separate test to measure the participant's perception of each pair of products and eliminate the influence of confounding factors. The test results revealed that apart from the difference in form, there is no difference between the uniqueness and attractiveness of two products in each pair.

Subsequently, the participants were instructed to indicate how accurate were the following statements in describing their feelings while recalling events: “I find it hard to relax,” “I am in a state of nervous tension,” “I feel that I am rather touchy,” and “I feel stressed” (39). All items used a seven-point Likert scale (ranging from 1 = strongly disagree to 7 = strongly agree). The scores were averaged to form an overall stress index (α = 0.86).

Finally, participants were instructed to indicate the extent to which they agreed on a seven-point Likert scale, with four statements measuring the escape motivation provoked by the event they had described. The statements were as follows: “I want to get rid of some dutched-up feelings,” “I would attempt to release or reduce some built-up tensions,” “I want to have my mind move at a slower pace” and “I want to give my mind a rest” (40). The statements were averaged to form an index of participants' escape motivation (α = 0.78).

### 4.3. Results

#### 4.3.1. Manipulation check

We first tested whether the manipulation of stress was successful. The results revealed that the manipulation caused increased stress levels in participants: those who recalled stressful events reported significantly higher feelings of stress than those in the control condition [M_stress_ = 4.35, M_control_ = 3.42, *t*_(100)_ = 3.491, *p* = 0.001]. This finding confirms that the stress manipulation was effective.

#### 4.3.2. Consumer preference

**Figure 2:**
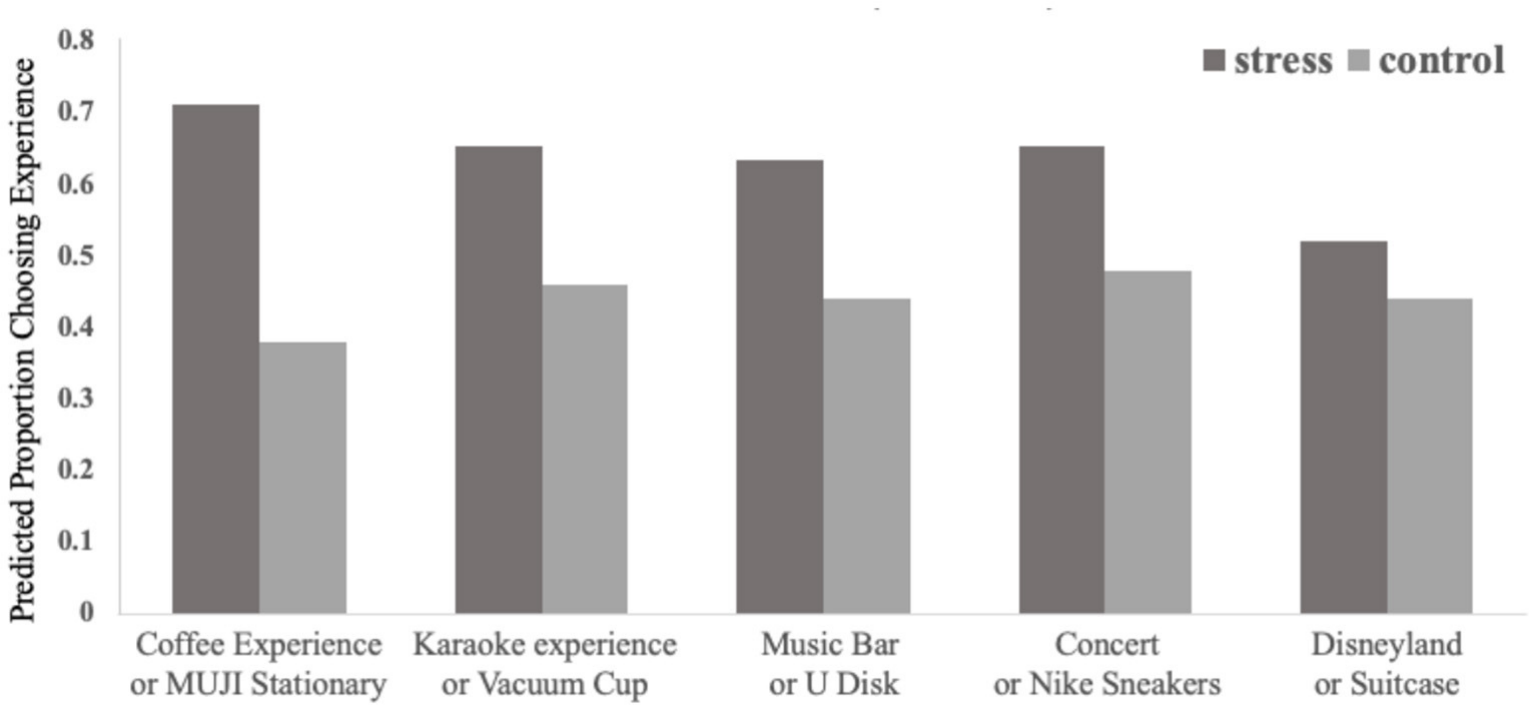
Proportion of participants choosing the experiential option, across five replicates, by condition (study 2).

We summed the number of experiential products that each participant chose (for a possible score from 0 to 5) and coded this number as each participant's relative preference for experiential goods vs. material goods. As predicted, participants in the stress condition showed greater preference for experiential goods than those in the control condition [M_stress_= 3.17, M_control_ = 2.20, *t*_(100)_ = 4.285, *p* < 0.001]. Figure 2 shows participants' preference for the experiential good in each pair at different conditions. Therefore, Study 2 replicated the results of Study 1.

#### 4.3.3. Mediation analysis

As predicted, participants in the stress condition were more motivated to escape than those in the control condition [M_stress_ = 5.00, M_control_ = 4.21, *t*_(100)_ = 2.607, *p* = 0.011], and self-escape motivation significantly predicted preference for the experiential goods [β = 0.339, *t*_(100)_ = 3.60, *p* < 0.001]. This suggests that self-escape motivation may play a mediating role in the effect of stress on preference for experiential purchases. To verify the mediating effect of self-escape motivation between stress and the preference for experiential consumption, we employed a bootstrapping procedure (41). This procedure computed a 95% confidence interval (CI) for the indirect and direct effects through 5,000 sampling. If a CI does not include 0, it indicates that the effect is significant. Following the approach suggested by Hayes (42), participants' preferences served as the dependent variable, the stress condition (coded −1 = control, 1 = stress) was included as the independent variable, and mean-centered motivation to escape was the mediating variable. The results revealed that self-escape motivation mediates the positive relationship between stress and preference for experiential goods [Effect = 0.0797, SE = 0.0394, 95% CI: (0.0205, 0.1853)].

### 4.4. Discussion

The results of study 2 showed that consumers who are highly stressed have an increased preference for experiences over material goods and this effect was mediated by a heightened motivation to escape from the psychological burden. Thus, Study 2 provides causal evidence that stress motivates individuals to escape, which subsequently increases their consumption preference for experiences over material possessions. While experiences facilitate the escape from psychological burdens rather than material possessions in most cases, there are some exceptions. This suggests a clear boundary condition for the effect. Study 3 will test this proposed boundary condition.

## 5. Study 3

Study 3 employed a 2 (stress: stress vs. control) ^*^ 2 (experiences: high cognitive resource–demanding vs. low cognitive resource–demanding) between-subjects design. We predicted that stress would not increase participants' preference for high cognitive resource–demanding experiential options as they cannot help consumers escape from the psychological burden caused by stress.

### 5.1. Participants

The participants comprised 209 undergraduate students recruited from a large university in China (101 males, 108 females; M_age_ = 19.34). Each participant received 10 RMB as compensation.

### 5.2. Procedure and stimuli

Study 3 was conducted in the lab. When the participants arrived, they were asked to take part in two independent experiments: first, a recall task testing their memory, second, a product selection task. The participants were blind to the aims of both tasks. We manipulated stress using the same procedure used in Study 2. After the event recall task, participants were shown three pairs of products, each consisting of one “experience” and one “material good.” Subsequently, they were instructed to indicate their preferences between the material good and experience. All participants were presented with the same material good (e.g., MUJI and Vacuum cup), while the experiential options were different. Some participants were presented with a low cognitive resource–demanding experiential option (e.g., Karaoke bar), whereas others were shown a high cognitive resource–demanding experiential option (e.g., Oil painting experience). In addition, as a manipulation check, we questioned participants to what extent they felt stressed (scores ranged from 1 = Not at all to 7 = Very much) and their perceptions of cognitive resource demands of the experiential options (1 = Very low, 7 = Very high). Finally, participants completed the demographic questions.

### 5.3. Results

#### 5.3.1. Manipulation check

We initially tested whether the manipulation of stress and experiential options had been successful. The results revealed that participants who recalled stressful events reported significantly higher feelings of stress than those in the control condition [M_stress_ = 5.05, M_control_ = 3.85, *t*_(207)_ = 7.277, *p* < 0.001]. The results also revealed that high cognitive resource–demanding experiential options were perceived to have a higher demand for cognitive resources than low cognitive resource–demanding experiential options [M_high cognitive resource − demanding condition_ = 4.01, M_low cognitive resource − demanding condition_ = 2.21, *t*_(207)_ = −10.928, *p* < 0.001], which, in turn, confirms that the manipulation of stress and experiential options were effective.

#### 5.3.2. Preference

**Figure 3:**
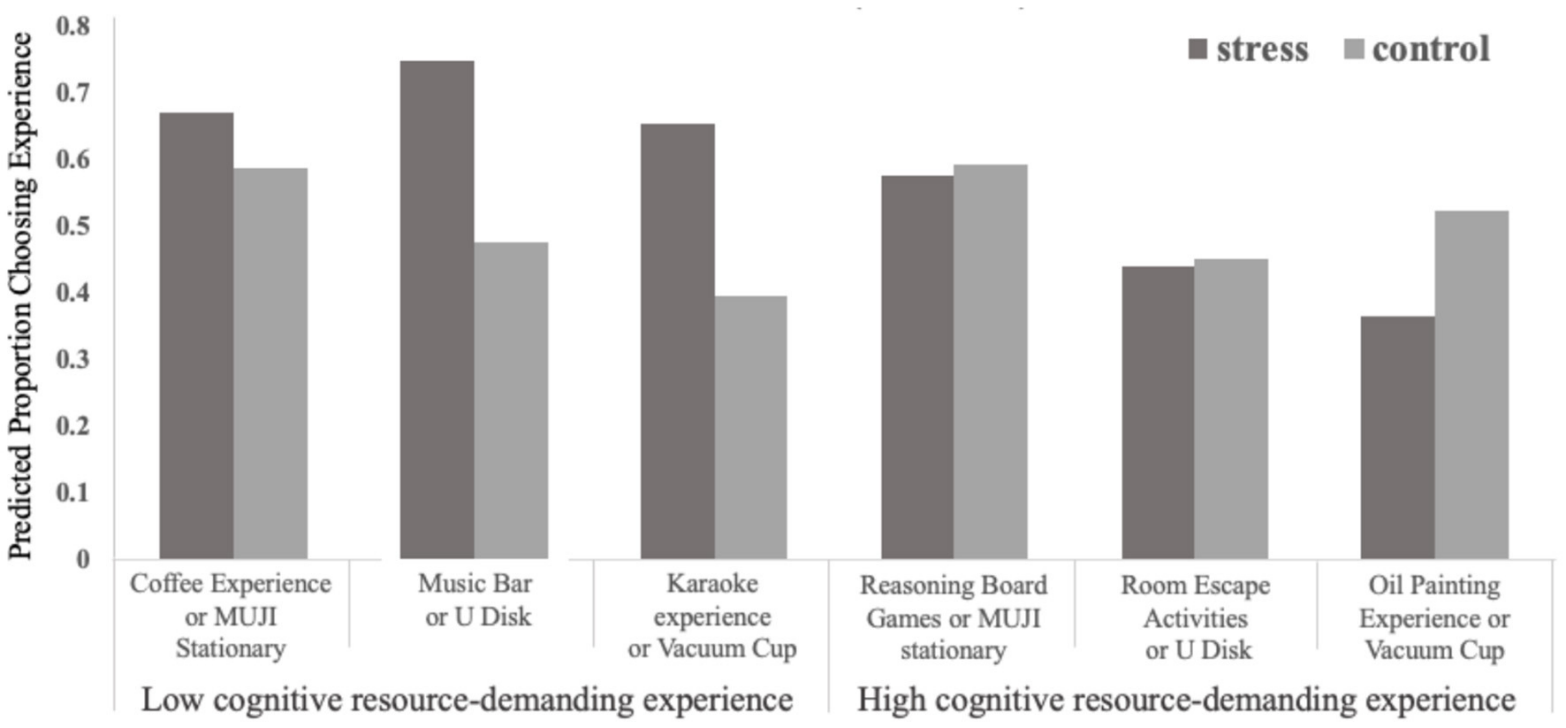
Proportion of participants choosing the experiential option, across five replicates, by condition (study 3).

Next, we summed the number of experiential products that each participant chose (for a possible score from 0 to 3) and coded this number as each participant's relative preference for experiential goods vs. material goods. A univariate analysis of general linear models revealed a significant interaction of stress and type of experiences on participants' preference [*F*_(1, 205)_ = 12.649, *p* < 0.001]. In the low cognitive resource–demanding experiential condition, stressed participants demonstrated an increased preference for the experiential options compared to those who were not stressed [M_stress_ = 2.08, M_control_ = 1.46, *F*_(1, 205)_ =16.18, *p* < 0.001], thus replicating previous results. However, in the high cognitive resource–demanding experiential condition, there was no significant difference in the preference for experiential options between stressed participants and non-stressed participants [M_stress_ = 1.39, M_control_ = 1.55, *F*_(1, 205)_ =1.45, *p* > 0.05]. The results are shown in Figure 3.

### 5.4. Discussion

The results of study 3 showed that compared with experiences with low cognitive resource demands, the effect of stress on consumption preference for experiences disappears when such experiences have high demands for cognitive resources. Study 3 revealed an important boundary condition: participants with stress were more likely to prefer experiencing options only if those experiences helped them escape the psychological burden of stress. However, when experiences were complicated and cognitively demanding, the effect of stress on experiences disappeared.

## 6. General discussion

Across one survey and two experiments, we demonstrate the effect of stress on individuals' relative preference for experiential vs. material consumption and its psychological mechanism. Our studies show that stress increases consumers' preference for experiences over material goods. Motivation to escape from psychological burden mediates the effect of stress on consumers' preference for experiences. Specifically, stress as a self-threat triggers individuals' motivation to escape from negative self-concept, and experiences can help individuals temporarily escape from negative self-recognition and provide more leisure value than material possessions. Therefore, individuals increase their consumption preference for experiences. In addition, only the positive process of relaxation drives the effect of stress on consumer preference for experience. In this case, stressed consumers' increased preference for experiential consumption is reduced when experiential consumption is complicated and requires significant mental resources.

### 6.1. Theoretical contribution

This research provides references for the study of public mental health by exploring the relationship between stress and individuals' consumption preferences for experiences and material possessions. An important innovation of research is to connect stress with experiential and material consumption, Existing studies have predominantly focused on the effects of stress on individuals' psychology and physiology; however, there is limited information on how stress affects consumer behavior. Consumption has become an important coping mechanism for stress and an avenue to enjoy leisure. Therefore, focusing on the relationship between stress and consumer behavior is necessary. Using self-escape theory, we have verified that stress increases individuals' consumption preferences for experiences over material possessions.

Our research also make theoretical contributions to research in the fields of psychology and consumer behavior. First, we provide direct empirical evidence that self-escape motivation can increase individuals' preference for experiences. Previous studies have determined that strong negative behaviors, such as suicide, binge eating, and gambling (21, 27), are common escape mechanisms. Furthermore, this study deduced that the consumption of experiences can also help individuals escape. This approach is more peaceful and positive and may improve individuals' ability to cope with difficulties. Therefore, experiential consumption is regarded as a happy escape, which helps us deepen the exploration of the escapism theory.

In the field of consumer behavior, enriching the research on antecedent variables of experiential and material consumption is also a major breakthrough in this study. Existing research has primarily focused on the difference and consequences of experiential and material consumption. However, our research introduces stress as the antecedent variable, explores its influence on individuals' consumption preferences for experiences and material goods, and explains the internal reasons of consumers' preferences for experiences and material possessions. Therefore, the exploration of individuals' consumption preferences for experiences in this research can be regarded as a supplement to related research.

### 6.2. Practical implications

Our conclusion provides recommendations for public mental health and stress management. Currently, emerging research is investigating interventions that could resist negative moods and facilitate the recovery from stressful situations. Importantly, non-pharmacological treatments such as enhancing exercise have been shown to be effective in treating symptoms of major depression (43). Our research suggests that experiential consumption can be considered as a relatively positive coping mechanism for stress with general interventions. As experiential purchases can generate and sustain happiness (44), consumers who are stressed can alleviate negative emotions and cope with stress through experiential purchases to obtain happiness.

In addition, our conclusions provide marketing advice for merchants in the experience industry. As stress increases individuals' consumption preferences for experiences, marketers of experiential products can locate target groups and undertake marketing plans accordingly. For example, travel products are increasingly favored by high-stress groups, such as office workers; therefore, for travel products, marketers can target these groups as customers. Moreover, marketers can strive to attract target groups by emphasizing the escapism effect of experiential products in advertisements and other marketing channels.

### 6.3. Research limitations and future research directions

This study has several limitations and issues that warrant future research. First, we adopted the general definitions of stress without differentiating between long-term vs. short-term stress (6). As long-term stress and short-term stress may have different effects, future research could further examine the types of stress to determine varying preferences on experiences vs. material possessions. In addition, there are many ways to measure stress, future research can adopt more diverse ways to measure stress, e.g., Patient Health Questionnaire 9 (45). Second, experiential consumption can be used as a coping mechanism for stress; however, whether this approach can effectively relieve stress and negative emotions remains unclear. Future research can examine consumers' stress and emotional differences before and after experiential consumption for further analysis. Finally, this study explores the effects of general experiential consumption. In fact, experiential consumption with different connotations (e.g., impulsive experiential consumption) deserves to be considered. Future study can pay attention to this and conduct in-depth research.

## Data availability statement

The raw data supporting the conclusions of this article will be made available by the authors, without undue reservation.

## Ethics statement

Ethical review and approval was not required for the study on human participants in accordance with the local legislation and institutional requirements. The patients/participants provided their written informed consent to participate in this study.

## Author contributions

YZ and XJ devised the project, the main conceptual ideas, and proof outline. XJ and TZ collected the research data and revised the manuscript. YZ and JL analyzed the sequencing data and wrote the initial manuscript. All authors contributed to the article and approved the submitted version.
